# Evaluation of Muscle Proteins for Estimating the Post-Mortem Interval in Veterinary Forensic Pathology

**DOI:** 10.3390/ani13040563

**Published:** 2023-02-06

**Authors:** Giuseppe Piegari, Valeria De Pasquale, Ilaria d’Aquino, Davide De Biase, Giulia Caccia, Carlo Pietro Campobasso, Simona Tafuri, Valeria Russo, Orlando Paciello

**Affiliations:** 1 Department of Veterinary Medicine and Animal Production, University of Naples Federico II, 80137 Naples, Italy; 2 Department of Pharmacy, University of Salerno, 84084 Salerno, Italy; 3 Department of Experimental Medicine, University of Campania “Luigi Vanvitelli”, 80138 Naples, Italy

**Keywords:** veterinary forensic pathology, time since death, postmortem interval, forensic sciences

## Abstract

**Simple Summary:**

The delimitation of the postmortem interval (PMI) is a very important parameter in veterinary forensic medicine. Indeed, the PMI can provide law enforcement with useful information to correctly determine a temporal relationship between a suspect’s actions and murder or to deny or confirm the testimony of a witness. Unfortunately, postmortem cadaveric changes, which are useful in investigating the PMI, have been poorly investigated in animals. In the present study, we evaluated the postmortem modifications of desmin and dystrophin over time and their correlation with PMI in dogs. To this aim, 10 dead adult dogs were evaluated for 4 days; for each animal, a cube of muscle tissue was collected from the vastus lateralis and triceps brachii. Muscle tissues were removed at 3 h postmortem and every 24 h until 96 h after death. Protein expression levels were analyzed by immunohistochemical examination and immunoblot analysis. The obtained results showed that dystrophin proteins had a higher degradation rate as compared to desmin. Overall, this study identified proteins with different postmortem degradation resistance, providing a reference basis for better investigating PMI in dogs.

**Abstract:**

Postmortem cadaveric changes are commonly used to estimate the postmortem interval (PMI) in humans and animals. However, these modifications have been poorly investigated in animals of interest to veterinary forensic pathology. The aim of this study was to investigate the potential use of muscle proteins (desmin and dystrophin) as biomarkers for estimating the PMI in dogs. For this study, 10 dead adult dogs were evaluated for 4 days in a temperature-controlled room at 19 ± 1 °C. For each animal, at 3, 24, 48, 72, and 96 h after death, a 1 × 1 × 1 cm cube of muscle tissue was removed from the vastus lateralis and triceps brachii. Protein expression levels were analyzed by immunohistochemical examination and immunoblot analysis. The obtained results showed rapid dystrophin degradation, with complete disappearance at 72 h after death. In contrast, desmin-positive fibers and desmin protein bands detected by immunoblot were observed on all 4 days of observation. Our findings suggest the potential use of muscle proteins as biomarkers for estimating the PMI in dogs.

## 1. Introduction

The postmortem interval (PMI) is defined as the time range between death and body examination [1]. It is considered one of the main research topics in forensic pathology. Indeed, the PMI can provide law enforcement with useful information to correctly determine a temporal relationship between a suspect’s actions and murder or to deny or confirm the testimony of a witness [2]. The PMI estimation is generally performed following the gross evaluation of the postmortem cadaveric changes, such as the cooling of the cadaver, ocular dehydration, muscle fiber rigidity and excitability, hypostasis development, and cadaver decomposition rate [3,4]. Normally, these changes have a well-defined order of progression; however, the rate of their onset and development is influenced by a wide range of variables that are both environmental and intrinsic to the subject itself, such as age [5], weight [6], size [7,8], previous pathology and cause of death [9], insect activity [10], temperature, and humidity [9,11]. Their application, precision, and accuracy are also strongly influenced by the phase of the PMI [12]. To overcome these limits, many research efforts have been devoted to standardizing additional techniques to estimate the PMI more accurately [13,14,15,16,17]. In recent years, postmortem protein degradation has been extensively investigated for its potential role in the delimitation of the PMI in humans [14,15,16,17]. Several studies have shown a correlation between protein degradation and time of death [14,15,16,17]. Furthermore, different proteins have been associated with different rates and patterns of degradation, suggesting that proteins have different resistance levels to postmortem proteolysis [18]. Among the assessed tissues, skeletal muscle has been identified as a promising candidate to estimate the PMI [19,20]. Skeletal muscle is the most abundant tissue in animal and human bodies, totally covered by the skin but also easily accessible during a forensic autopsy [21]. It is also more resistant to postmortem cadaveric changes than other organs like the liver and kidney [22]. In human forensic pathology, postmortem skeletal protein degradation has been investigated using both case-based studies on human cadavers and experimental works on animal models, such as mice [23], rats [24,25,26], and pigs [27]. In contrast, in veterinary forensic pathology, so far, no studies have been conducted to investigate postmortem protein degradation in dogs (*Canis familiaris*). Dogs are some of the few animals that live in close contact with humans, in both urban and rural contexts [28]. Therefore, they are commonly victims of abuse and unlawful killings [29]. Dogs also represent one of the main species involved in illegal fighting [30] and animal–vehicle collisions [31]. All these features have made dogs a main species of veterinary forensic interest. However, few reliable methods for investigating time since death in dogs have been reported in the literature. Although studies conducted on human and animal models have provided valuable information in veterinary forensic sciences, the physical and physiological differences between species do not allow for the direct application of assessed parameters to dogs and, more generally, to the broad range of animals of veterinary forensic interest. In this field, we investigated the postmortem morphological and biochemical modifications of triceps brachii and vastus lateralis skeletal muscles, both considered among the largest muscles of the animal body, which are well protected by the skin and bones but also relatively distant from the thoraco-abdominal organs. Therefore, they are expected to be less affected by the thanatomicrobiome activities during the first postmortem cadaveric decomposition phases. In light of these observations, the aims of this study were to (1) investigate the time-dependent histological changes of dog muscle tissues over time; (2) assess the postmortem degradation of the desmin and dystrophin proteins in dog skeletal muscles via immunohistochemical examination and Western blot analysis and (3) standardize an immunohistochemical and Western blot laboratory panel to investigate the time since death in dogs.

## 2. Materials and Methods

### 2.1. Muscle Sample Collection

Ten dead mixed-breed dogs (five male and five female), aged between 6 and 12 years, were recruited into the study. None of the studied dogs showed any evidence of neuromuscular or metabolic diseases including hypothyroidism, hyperadrenocorticism, renal disease, diabetes mellitus, and neoplasia. Immediately after death, animals were maintained at controlled temperature ranging between 18 and 20 °C for 4 days at the Section Room of the Department of Veterinary Medicine and Animal Production of the University of Naples Federico II. For each animal, at 3, 24, 48, 72, and 96 h after death, a 1 × 1 × 1 cm cube of muscle tissue was removed from the vastus lateralis and triceps brachii at a depth of 1 cm. The samples were then divided into five groups on the basis of the time elapsed since death: Group A comprised 20 samples (10 samples from the triceps brachii muscle and 10 samples from the vastus lateralis muscle) taken 3 h after death. Group B comprised 20 samples (10 samples from the triceps brachii muscle and 10 samples from the vastus lateralis muscle) taken 24 h after death. Groups C, D, and E comprised 20 samples each (10 samples from the triceps brachii muscle and 10 samples from the vastus lateralis muscle) taken at 48, 72, and 96 h after death, respectively. Samples were divided into two aliquots. Aliquots for histopathological examination and immunohistochemical analysis were frozen in isopentane that was pre-cooled in liquid nitrogen, and then they were stored at −80 °C until further processing. Aliquots for Western blot analysis were stored at −80 °C.

### 2.2. Sampling Procedure

All samples were collected after a 1 cm skin incision. The distance between each sampling point was at least 2 cm. Furthermore, samples were taken in rigorous asepsis conditions using sterile instruments and transported to the laboratory of the Department of Veterinary Medicine and Animal Production of the University of Naples Federico II within 5 minutes. Collected samples were frozen within 15 minutes.

### 2.3. Histology and Immunohistochemical Examination

Frozen sections (8 µm thick) were stained according to our routinely performed laboratory staining procedure [32]. Specifically, the following stains were performed: (1) hematoxylin and eosin (HE) for a basic morphologic evaluation, and (2) periodic acid Schiff (PAS) to evaluate the glycogen storage in muscle tissue [33,34]. For immunohistochemistry (IHC), frozen sections (8 µm thick) were processed with the MACH1 Universal HPR Polymer Detection Kit (BioCare Medical LLC, Concord, CA, USA). Briefly, the sections were dried for 1 h at room temperature and fixed in acetone at 4 °C for 3 min; a peroxide block was applied for 15 min at room temperature, and then the sections were incubated for 30 min with a background sniper (BioCare Medical LLC). The sections were incubated overnight at 4 °C with the following primary antibodies:

- Mouse anti-*DYS-1* monoclonal antibody (NCL-DYS1) (NovoCastra Laboratories Ltd, Newcastle, UK) directed against the ROD domain of the muscular dystrophin protein;
- Mouse anti-*DYS-2* monoclonal antibody (NCL-DYS2) (NovoCastra Laboratories Ltd, Newcastle, UK) directed against the C-terminal domain of the muscular dystrophin protein;
- Mouse anti-desmin clone D33 monoclonal antibody (M0760) (DakoCytomation, Denmark).

The reaction was revealed by using 3,3’-diaminobenzidine (DAB) chromogen diluted in a DAB substrate buffer. Finally, sections were counterstained in Carazzi’s hematoxylin. 

The percentage of muscle fibers with cytoskeletal positivity to dystrophin 1, dystrophin 2, and desmin was scored as follows: >90% positively stained fibers (score: 4), 90–60% positively stained fibers (score: 3), 60–30% positively stained fibers (score: 2), 1–30% positively stained fibers (score: 1), and negative staining observed in the fibers of the section (score: 0).

### 2.4. Western Blotting

Selected samples (triceps and quadriceps) were mechanically homogenized in a lysis buffer containing 50 mM Tris HCl pH 7.5, 150 mM NaCl, 1 mM EDTA, 1 mM EGTA, 10% glycerol, 1% Triton X-100, 1 mM β-glycerophosphate, 1 mM phenylmethylsulfonyl fluoride, a protease inhibitor cocktail tablet, 1 mM sodium orthovanadate, and 2.5 mM sodium pyrophosphate [35,36]. Homogenates were incubated for 30 min on ice, and the supernatants were collected and centrifuged for 30 min at 14,000× *g*. Protein concentration was estimated with a Bradford assay, and 50 μg/lane of total proteins were separated on SDS polyacrylamide gels and transferred to nitrocellulose membranes [37,38]. Membranes were treated with a blocking buffer (25 mM Tris HCl, pH 7.4, 200 mM NaCl, and 0.5% Triton X-100) containing 5% non-fat powdered milk for 1 h at room temperature. Incubation with the anti-dystrophin (DYS-1/DYS-2) and anti-desmin primary antibodies was carried out overnight at 4 °C. After washing, membranes were incubated with the HRP-conjugated secondary antibody (sc-2031, Santa Cruz Biotechnology Inc, Dallas, TX, USA) for 1 h at room temperature, and then, the proteins were visualized by enhanced chemiluminescence (ECL) (RPN2232SK, Amersham, GeHealthcare, UK). In addition, the following primary antibodies were used to monitor equal protein loading of protein in all gel lanes:

- Mouse anti-GAPDH monoclonal antibody (clone 6C5, sc-32233, Santa Cruz Biotechnology Inc, Dallas, TX, USA);
- Mouse anti-alpha-actinin (clone *H-2*, sc-*17829*, Santa Cruz Biotechnology Inc, Dallas, TX, USA).

Band intensities were quantified on scanned images using Image J 1.53 software (National Institute of Health). Samples from Group A (3 hpm) were considered to contain the native form of the protein, and all bands of samples from the other groups were compared with them.

### 2.5. Statistical Analysis

The SPSS 20.0 package (SPSS Inc., Chicago, IL, USA) was used for statistical analysis of the data. The Mann–Whitney test, a nonparametric test for two independent samples, and Student’s t-test were used to assess the differences between groups. *p* values < 0.05 were considered statistically significant. The associations between the percentage of positive fibers and the time since death was evaluated by Spearman’s Rho correlation. 

## 3. Results

### 3.1. Histopathological Examination

**Figure 1:**
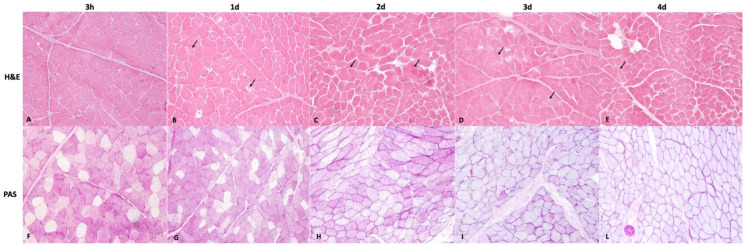
Representative sections from muscle tissues at different times since death. (**A**) Section from muscles taken at 3 hpm (Group A) showing no microscopical changes; (**B**) section from muscle taken at 1 day postmortem (Group B) showing focal areas of autolysis (arrows). (**C–E**): Section from muscles taken at 2, 3 and 4 days postmortem (Groups C-D-E), respectively. Sections showing only small focal or multifocal areas of autolysis (arrows) (H&E, original magnification 20×). (**F**) Section from muscles taken at 3-hpm (Group A) showing strong PAS-positive fibers; (**G**) section from muscle taken at 1 day (Group B) postmortem showing PAS-positive fibers. (**H**) Section from muscles taken at 2 days (Group C) postmortem showing a moderate number of PAS-positive fibers. (**I**) section from muscles taken 3 days (Group D) postmortem showing a low number of mildly PAS-positive fibers. (**L**) Section from muscles taken 4 days postmortem (Group E) showing no PAS-positive fibers. (Periodic acid shiff (PAS) staining, original magnification 20×).

All 3 h postmortem samples (Group A) stained with H&E showed intact muscle fibers and no microscopic changes in both the triceps brachii and vastus lateralis muscles. One-day postmortem samples (Group B) showed small focal areas of autolysis in 7 out of 10 cases in triceps brachii muscles and in 6 out of 10 cases in vastus lateralis muscles (Figure 1B). In the samples at 2 (Group C), 3 (Group D) and 4 (Group E) days postmortem, we observed mild reduction in cell–cell adhesion or only small focal or multifocal areas of autolysis, characterized by destruction of fibers and the loss of cell borders (group C: 8 out of 10 cases in triceps brachii muscles and 8 out of 10 cases in vastus lateralis muscles; groups D and E: 10 out of 10 cases in triceps brachii muscles and 10 out of 10 cases in vastus lateralis muscles) (Figure 1C–E). The PAS stain was also strongly positive in the cell cytoplasms at 3 h and 1 day postmortem (Group A and B), while a variable number of mildly positive fibers were still detectable at 2 (Group C) and 3 (Group D) days postmortem. No PAS-positive fibers or a low number of PAS-positive fibers were also observed in all samples at 4 days postmortem in both triceps brachii and vastus lateralis muscles (Figure 1F–L). 

### 3.2. Immunohistochemical Examination

**Figure 2:**
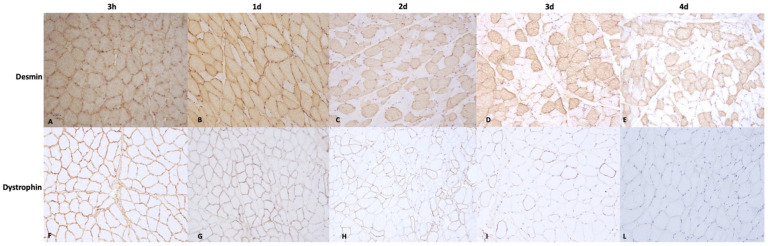
Immunohistochemical staining of desmin and dystrophin at different times since death. (**A**) Section from muscles taken at 3 hpm (Group A) showing >90% (score 4) desmin-positive fibers; (**B**) section from muscle taken at 1 day postmortem (Group B) showing >90% (score 4) desmin-positive fibers in the section; (**C**) section from muscles taken at 2 days (Group C) postmortem showing desmin positivity ranging between 60–90% (score 3). (**D**) Section from muscles taken at 3 days postmortem (Group D) showing 30–60% (score 2) desmin-positive fibers. (**E**) Section from muscles taken at 4 days postmortem (Group E) showing 30–60% (score 2) desmin-positive fibers. (**F**) Section from muscles taken at 3 hpm (Group A) showing >90% (score 4) dystrophin-positive fibers; (**G**) section from muscle taken at 1 day (Group B) postmortem showing 60–90% (score 3) dystrophin-positive fibers in the section; (**H**) section from muscles taken at 2 days postmortem (Group C) showing that the number of positive fibers ranged between 30 and 60% (score 2). (**I**) Section from muscles taken at 3 days postmortem (Group D) showing 1–30% (score 1) dystrophin-positive fibers. (**L**) Section from muscles taken at 4 days postmortem (Group E) showing a total absence of dystrophin-positive fibers.

In 3 hpm samples (Group A), both desmin and dystrophin 1 and 2 immunoreactivity were observed in all the assessed samples, demonstrating >90% desmin- and dystrophin-positive fibers in both the triceps brachii and vastus lateralis muscles (score 4). In 1-day postmortem muscles (Group B), the majority of the assessed samples showed a percentage of desmin-positive fibers >90% (score 4) (triceps brachii: 7/10 samples; vastus lateralis: 8/10 samples) in both the triceps brachii and vastus lateralis muscles; a percentage of desmin-positive fibers ranging between 60 and 90% (score 3) was instead observed in a lower number of cases (triceps brachii: 3/10 samples; vastus lateralis: 2/10 samples). Furthermore, dystrophin immunopositivity was observed in 60–90% of fibers (score 3) in most assessed samples in group B (triceps brachii: 8/10 samples; vastus lateralis: 7/10 samples). The percentage of dystrophin-positive fibers at 1 day postmortem was also statistically lower than that observed in 3 h postmortem muscles (Group A vs. Group B; *p* < 0.05). The prevalence of desmin- and dystrophin-positive fibers in 2-day postmortem muscles (Group C) was as follows: 30–60% (score 2) dystrophin-positive fibers (triceps brachii: 6/10 samples; vastus lateralis: 6/10 samples) and 60–90% (score 3) desmin-positive fibers (triceps brachii: 8/10 samples; vastus lateralis: 8/10 samples). The Mann–Whitney U test also showed a statistically lower degree of dystrophin- and desmin-positive fibers than those observed in 3 h and 1-day postmortem samples (Group C vs. Group A and B; *p* < 0.05). In 3-day postmortem samples (Group D), muscles showed a percentage of dystrophins positive fibers ranged between 1 and 30% (score 1) (triceps brachii: 9/10 samples; vastus lateralis: 9/10 samples) or absence of immunopositivity (score 0) (triceps brachii: 1/10 samples; vastus lateralis: 1/10 samples); desmin was instead detected in 30–60% (score 2) of fibers (triceps brachii: 7/10 samples; vastus lateralis: 7/10 samples) in the majority of the assessed samples. At 4 days postmortem (Group E), all samples showed a total absence of immunopositivity to dystrophin (score 0) (triceps brachii: 2/10 samples; vastus lateralis: 2/10 samples) or a low percentage of dystrophin-positive fibers, ranging between 1 and 30% (score 1) (triceps brachii: 8/10 samples; vastus lateralis: 8/10 samples); in contrast, the majority of tissue samples showed an immunopositivity for the desmin protein, ranging between 30 and 60% (score 2) of fibers (triceps brachii: 8/10 samples; vastus lateralis: 7/10 samples) (Figure 2). In addition, the percentage of desmin- and dystrophin-positive fibers in 4-day postmortem samples was lower than that detected in the 2-day postmortem group (Group E vs. Group C; *p* < 0.05). 

**Figure 3:**
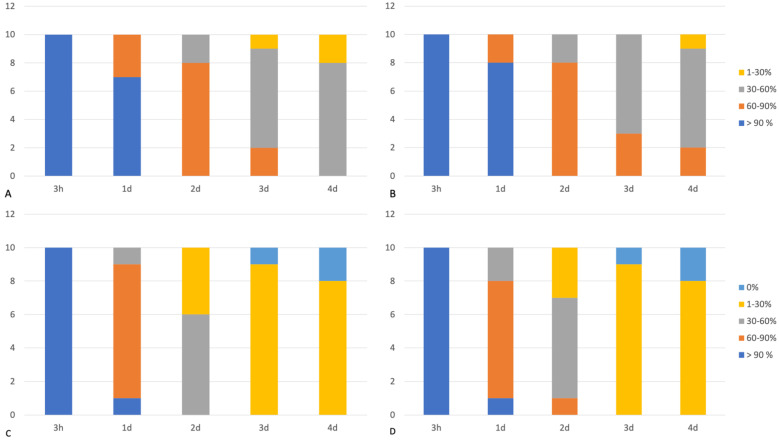
The percentage of positive fibers for each assessed group. (**A**) Desmin-positive fibers in triceps brachii muscles, (**B**) desmin-positive fibers in vastus lateralis muscles; (**C**) dystrophin-positive fibers on triceps brachii muscles; (**D**) dystrophin-positive fibers in vastus lateralis muscles.

Spearman’s Rho test also showed a negative significant correlation between desmin- and dystrophin-positive fibers and time since death. No statistical difference in dystrophin- and desmin-positive fibers was observed between the triceps brachii and vastus lateralis muscles in all assessed groups. Finally, the percentage of desmin-immunopositive fibers in groups B, C, D, and E was higher if compared with the percentage of dystrophin 1- and 2-immunopositive fibers in the same groups. The results of the immunohistochemical examination are summarized in Figure 3.

### 3.3. Western Blot Analysis

**Figure 4:**
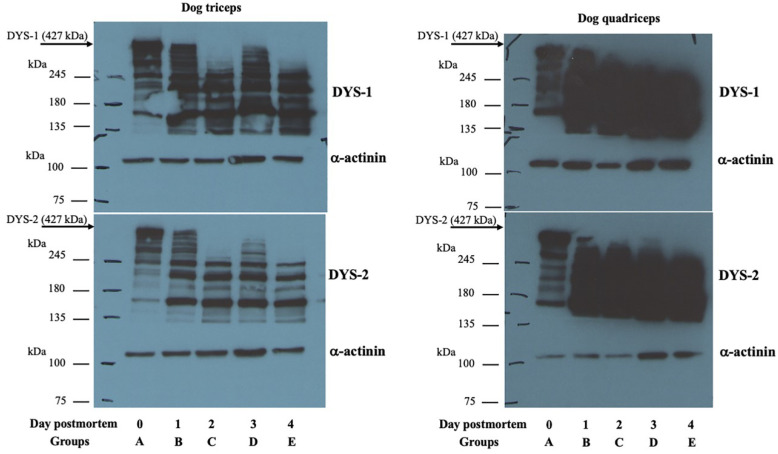
Representative image of the Western blotting analyses performed for detecting dystrophin protein in the dog muscle samples. Western blot analysis shows a rapid reduction of the native dystrophin band, with complete disappearance at 2 days postmortem. In contrast, α-actinin protein was observed in all examined groups. (**Left**) Dog triceps, (**Right**) Dog quadriceps.

The blotting profile in Figure 4 shows that both anti-DYS1 and DYS2 antibodies recognized the 427 kDa dystrophin protein in both the triceps brachii and vastus lateralis muscles from the muscle tissue of dogs at 3 h after death (Group A). Although the 427 kDa dystrophin band was still slightly visible in muscle tissue at 1 day postmortem (Group B), it completely disappeared at 2 (Group C), 3 (Group D) and 4 (Group E) days postmortem in all assessed cases. The α-actinin antibody showed a band at approximately 100 kDa that was observed in all the studied groups (Figure 4).

**Figure 5:**
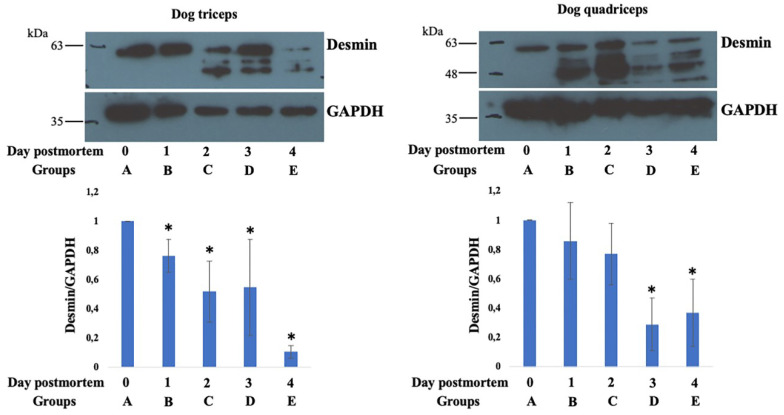
Representative image of the Western blotting analyses performed for detecting desmin and GAPDH protein levels. Desmin and GAPDH bands were present throughout the investigated periods. Densitometric analysis of the bands was performed, with results presented as the mean ± SD of four independent experiments of equal design for triceps and three independent experiments of equal design for quadriceps. * *p* < 0.05. (**Left**) Dog triceps, (**Right**) Dog quadriceps.

In addition, desmin protein bands at approximately 60 kDa were detectable in all examined groups. Statistical differences were observed among the examined groups (*p* < 0.05 vs. Group A), as shown in Figure 5. GAPDH was observed in all the studied groups and used to normalize the native desmin band intensity between the muscle samples analyzed. 

## 4. Discussion

The results of this study demonstrate a specific association between postmortem morphological changes and protein degradation in muscle tissue and time elapsed since death in dogs. On a qualitative basis, histological examination, which was conducted on muscle tissues until 4 days postmortem, allowed us to observe slight postmortem changes, primarily characterized by reduced fiber–fiber adhesion and focal or multifocal areas of autolysis. A progressive reduction in intra-cytoplasmatic PAS-positive deposits with disappearance at 96 h postmortem was also observed via PAS staining. Fiber autolysis is an inevitable consequence of proteases’ activities on human and animal tissues, while the progressive reduction in PAS-positive fibers reflects glycogen degradation, which is mainly caused by postmortem glycogenolysis [39,40]. Overall, the strong postmortem morphological preservation observed in our study agrees with published data from other authors. Similar results were indeed observed by Erlandsson (2007) [41], who reported the good preservation of dog myocardium until 7 days postmortem. Furthermore, the present results match with those reported by Tavichakorntrakool et al., (2008) [42], who detected histological changes, such as vacuolization and autolysis, in human muscles stored at 25 °C from 6 h after death. Unfortunately, a direct comparison between the aforementioned studies and our results is difficult due to the different storage temperatures used, which could inevitably influence the rate of the development of the postmortem changes in muscle tissue. With respect to the postmortem protein degradation, we demonstrated a rapid reduction in the dystrophins, with the complete disappearance of the DYS-1 and DYS-2 signals at 2 days postmortem. However, low numbers of immunopositive fibers were still detectable until 4 dpm by immunohistochemical examination. In addition, the Mann–Whitney U test showed statistically differences between groups and a negative correlation between time of death and protein degradation. Similarly, our findings showed a negative correlation between desmin protein degradation and time of death. However, desmin protein degradation was observed to be slower than that of dystrophin under both immunohistochemical and Western blot analysis. Taken together, these findings suggest a postmortem relationship between the PMI and protein degradation, as well as a difference in the rate of postmortem degradation between the two assessed proteins. Different rates of degradation among cellular proteins have been previously reported [18,43]. Indeed, the protein degradation rate appears to be strongly influenced by a broad range of intrinsic variables, such as protein structures, amino acid conformations, and post-translational modifications [18]. With respect to desmin and dystrophin proteins, they are both important components of the cytoskeletal structure of skeletal muscle and are united by a high sensitivity to calpain, proteasome, and lysosome activities [44]. However, they present important differences in conformation, location, and relative abundance. Dystrophin is a protein that links the cytoskeletal sarcoplasm to the extracellular matrix through a dystrophin-associated glycoprotein complex and plays an important role in the stabilization of the sarcoplasm in skeletal muscle [45]. However, dystrophin only represents 0.002% of total muscle proteins [45]. In contrast, desmin is the major component of the intermediate filament; it is located around the Z-disk of the sarcomere and links the Z-disk to the subsarcolemmal cytoskeleton. It is also more abundant than dystrophin, accounting for 0.35% of total muscular proteins [45]. Thus, the differences in protein abundance between these two assessed molecules could be considered additional contributing factors to the higher degradation rate of the dystrophin protein compared to that of the desmin protein. Overall, our findings are consistent with previously published studies conducted on pigs [19] that showed a persistence of the desmin protein for several days after death (up to 224.0 hpm). Similarly, our results agree with those reported by Wojtysiak and Górska (2018) [46], who detected the rapid degradation of dystrophin protein in turkey breast muscles. However, a direct comparison of the results is not possible due to differences in temperature storage between our study (19 °C) and the abovementioned research (4 °C). Finally, regarding α-actinin and GAPDH, no differences in protein levels were observed among the assessed groups, suggesting that these proteins have a higher resistance to postmortem proteolysis than do dystrophin and desmin. A previous study revealed the high resistance of α-actinin up to 210 hpm in pig muscles [19]. Similarly, a study conducted on rat skeletal muscles showed the resistance of GAPDH up to 96 h postmortem [47]. α-actinin and GAPDH are two of the main proteins commonly used in Western blot analysis to confirm the equal loadings of proteins in all lanes. However, the high resistance of α-actinin and GAPDH to postmortem proteolysis encourages their use as control markers even in protein degradation studies until the 4th day postmortem in dogs. 

## 5. Conclusions

The estimation of the PMI is a challenge in both human and veterinary forensic pathology. The range of temperatures applied in the present study (19 °C) allowed us to identify proteins with different rates of degradation, providing a reference basis for better investigating different PMI phases in dogs. Specifically, the slow postmortem degradation of desmin suggests the potential for its use as a biomarker to assess the late postmortem phase. Furthermore, the rapid postmortem degradation of dystrophin suggests its validity as a biomarker to investigate the early postmortem phase in dogs. Finally, the higher resistance of GAPDH and α-actinin to postmortem proteolysis encourages their use as control markers in Western blot analysis up to 4 dpm. Further studies will be needed to investigate the postmortem muscle modifications at different environmental temperature ranges and over longer PMIs in dogs.

## Data Availability

All relevant data are listed in the manuscript.
